# Phylogenetic community structure: temporal variation in fish assemblage

**DOI:** 10.1002/ece3.1026

**Published:** 2014-05-01

**Authors:** Sergio Santorelli, William Magnusson, Efrem Ferreira, Erica Caramaschi, Jansen Zuanon, Sidnéia Amadio

**Affiliations:** 1 Programa de Pós graduação em Biologia de Água Doce e Pesca Interior, Instituto Nacional de Pesquisas da AmazôniaManaus, Brazil; 2 Centro de Estudos Integrados da Biodiversidade AmazônicaManaus, Brazil; 3 Coordenação de Pesquisas em Biodiversidade, Instituto Nacional de Pesquisas da AmazôniaManaus, Brazil; 4 Programa de Pós graduação em Ecologia, Universidade Federal do Rio de JaneiroRio de Janeiro, Brazil

**Keywords:** Competitive exclusion, environmental filter, local scale, phylogenetic patterns, temporal scale

## Abstract

Hypotheses about phylogenetic relationships among species allow inferences about the mechanisms that affect species coexistence. Nevertheless, most studies assume that phylogenetic patterns identified are stable over time. We used data on monthly samples of fish from a single lake over 10 years to show that the structure in phylogenetic assemblages varies over time and conclusions depend heavily on the time scale investigated. The data set was organized in guild structures and temporal scales (grouped at three temporal scales). Phylogenetic distance was measured as the mean pairwise distances (MPD) and as mean nearest-neighbor distance (MNTD). Both distances were based on counts of nodes. We compared the observed values of MPD and MNTD with values that were generated randomly using null model independent swap. A serial runs test was used to assess the temporal independence of indices over time. The phylogenetic pattern in the whole assemblage and the functional groups varied widely over time. Conclusions about phylogenetic clustering or dispersion depended on the temporal scales. Conclusions about the frequency with which biotic processes and environmental filters affect the local assembly do not depend only on taxonomic grouping and spatial scales. While these analyzes allow the assertion that all proposed patterns apply to the fish assemblages in the floodplain, the assessment of the relative importance of these processes, and how they vary depending on the temporal scale and functional group studied, cannot be determined with the effort commonly used. It appears that, at least in the system that we studied, the assemblages are forming and breaking continuously, resulting in various phylogeny-related structures that makes summarizing difficult.

## Introduction

Understanding species coexistence is one of the central objectives of ecology and has been debated for over a century. Hypotheses related to competition (e.g., Darwin 1859; Gause 1934; Elton 1946; Diamond 1975), predation (e.g., Paine 1966), abiotic factors (e.g., Andrewartha and Birch 1954; Dunson and Travis 1991; Weiher and Keddy 1995), and processes associated with dispersal limitation (e.g., MacArthur and Wilson 1967; Connor and Simberloff 1979; Bell 2000; Hubbell 2001) have frequently been discussed in the literature (e. g. Clark 2012; Halley and Iwasa 2012; Rosindell et al. 2012). However, there is debate as to whether species composition in local assemblies is mainly determined by random, deterministic, or historical factors (Clements 1916; Gleason 1926; Ricklefs 1987; Cornell and Lawton 1992).

Hypotheses about phylogenetic relationships among species allow inferences about the mechanisms that most affect species coexistence. This approach was first used by Darwin (1859) and is now widely accepted. According to Darwin, species of the same genus usually have similar habits, and competition between these species for particular resources will be stronger than between species of different genera, and this will limit coexistence of congeneric species. Elton (1946) found that competition could explain the difference in the frequency of congeneric species found in the same locality and showed that this effect resulted in a strong tendency for species of the same genus to occur in different habitats.

Webb et al. (2002) suggested that species composition is not random with respect to phylogenetic relatedness due to environmental filtering and competitive exclusion. According to their hypothesis, phylogenetic clustering (“phenotypic attraction”) of species in an assemblage is determined by the action of environmental filters. That is, the use of habitat is determined by ecological characteristics shared with phylogenetically closely related species. Phylogenetic overdispersion (“phenotypic repulsion”) of species can result when taxa that are closely related phylogenetically are also more similar in resource use and tend to exclude each other locally, such that there is minimal overlap between the resource use of coexisting species (competitive exclusion), or when phylogenetically distant taxa converge in the use of resources and are favored under the same environmental conditions. However, Webb et al. (2002) noted that the repulsion of convergent ecological phenotypic characteristics can result in an assembly composition that appears phylogenetically random.

The action of the processes described by Webb et al. (2002) is the probable cause of the phylogenetic pattern that has been observed for several taxonomic groups under different environmental conditions (Helmus et al. 2007; Newton et al. 2007; Edwards and Zak 2010; Kamilar and Guidi 2010; Machac et al. 2011; Parras et al. 2011; Rabosky et al. 2011; Merwin et al. 2012). However, other possible interactions should also be evaluated to explain patterns of phylogenetic similarity in assemblages (e.g., Weiblen et al. 2006; Vamosi and Vamosi 2007; Cadotte et al. 2010; Letcher 2010; Liu et al. 2012).

Conclusions about the interactions between species based on the composition of assemblages also depend on the scale being investigated (Levin 1992; McGill 2010; Shipley et al. 2012). As the spatial scale, taxonomic level, and decisions about guild membership influence conclusions about the effects of evolutionary and ecological processes in the phylogenetic structure of assemblies, it is important to determine at which scales species are clustered or dispersed phylogenetically (Cavender-Bares et al. 2006; Swenson et al. 2006, 2007). Nevertheless, most studies have been short term and assume that phylogenetic patterns identified are stable over time.

Ecological conclusions are based on the apparent associations of a subsample of species that are subject to a particular set of sampling techniques that are assumed to represent some conceptual assemblage that exists in nature. However, all sampling techniques involve some bias (Gotelli and Colwell 2001). Also, competitive interactions are only expected for species within the same guild. Therefore, results will depend on decisions as to which species to include in analyses, and the relative susceptibility of these species to capture.

Co-occurrence also has a temporal aspect for animals. While turnover in sessile plants within a spatial sampling unit, such as a plot or lake, may be relatively slow, animals may move into and out of sampling units at frequent intervals. This does not mean that they do not affect each other because there may be residual effects. One species may reduce a resource for another that arrives after the first has left, and individuals may learn to avoid landscape elements that are frequently used by a competitor even when that competitor is not present.

In this study of fish assemblages in a single site over 10 years, we show that the structure in phylogenetic assemblages varies over time and conclusions depend heavily on the time scale investigated. Therefore, it is important not only to identify patterns, but also indicate the scales of time and space over which they act. It appears that, at least in the system that we studied, the assemblages are forming and breaking continuously, resulting in various phylogeny-related structures that makes summarizing difficult.

## Material and Methods

### Data source

The data were generated in a long-term project undertaken by the Research Group on Ecology and Conservation of Freshwater Fish at the National Institute of Amazonian Research – CBIO/INPA. Fish were captured monthly in Catalão Lake, a floodplain lake, located at coordinates 3°10′04″S and 59°54′45″W, during 10 years (for more details of study area and sampling methods see Appendix S1).

### Guild classification

The data set was initially organized in different guild structures and temporal scales. The first taxonomic group we call “Overall Assemblage”. This group includes 151 species for which we had phylogenetic information. Six other groups (piscivorous: 23 spp, carnivorous: 11 spp, invertivorous: 24 spp, herbivorous: 15 spp, detritivorous: 23 spp, and omnivorous: 33 spp) were established according to their similarity in diet based on literature records (Appendix S2). Piscivorous species feed mainly on other fish, ingested in pieces or whole; carnivorous species had a diet based mainly on animal prey without predominance of any specific group; invertivorous species eat mainly invertebrates; herbivorous species eat mainly plant material; omnivores consumed plant and animal foods in similar quantities; and detritivorous species ate mainly detritus. The species and their feeding habits are given in Appendix S3.

### Temporal scale

Capture data for the 10 years were grouped at three scales, to focus on different aspects of seasonality. In the first temporal scale (sample), we consider captures within each month as sample units, giving a total of 117 observations over 10 years. At this scale, individuals within a sample unit were physically present together. In the second temporal scale (calendar months), we grouped all samples of each calendar month sampled over time, generating 12 sampling units representing 10 years of sampling (each month had data accumulated over 10 years). At this scale, species that use the same seasonal resources in the lake are potentially present together. For the third temporal scale (years), we combined the 12 months of each year as a sample unit, generating 10 observations. At this scale, species that have the same long-term temporal trends are potentially present together.

### Data analysis

All analyses were carried out separately for the whole assemblage and each functional group for all temporal scales in the R software (Development Core Team 2012).

#### Phylogenetic structure

We constructed a phylogenetic hypothesis about all species based on data from Vari (1984, 1989); Walsh (1990); Malabarba et al. (1998); Reis (1998); Castro and Vari (2004); Moyer et al. (2004); Piza (2007) and Mirande (2010). Phylogenetic distance was measured as the mean pairwise distances (MPD) between species and as mean nearest-neighbor distance (MNTD) between each species and its closest relative based on counts of nodes that separate species (Webb 2000) using the R package “picante” (Kembel et al. 2010).

To determine whether the phylogenetic structure of the whole assemblage and of the functional groups is different from what would be expected by chance over time, we compared the observed values of MPD and MNTD with values that were generated randomly using a null model (Gotelli and Graves 1996) as follows:


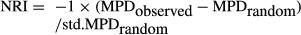



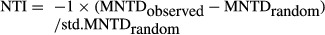


The NRI (Net Relatedness Index) considers the entire phylogenetic tree, and NTI (Nearest Taxon Index) considers only relatedness to the closest taxon, MPD_random,_ and MNTD_random_ are values of these statistics derived from different permutations of species within the phylogenetic hypothesis, and std.MPD_random_ and std. MNTD_random_ are standard deviations of the values 10000 MNTD_random_ and MPD_random_, respectively. Positive values of NRI and NTI indicate that the species that make up the assembly, or functional group, are more phylogenetically related (phylogenetically clustered) than would be expected by chance, while negative values indicate that the species are phylogenetically more distant (phylogenetic overdispersion). To assess how these indices are different from what would be expected by chance, we used the null model independent swap (Gotelli and Entsminger 2003).

#### Randomness test

The serial runs test (Zar 1999) was used to assess the temporal independence of the NRI and NTI indices over time. The null hypothesis for the test was that the distribution of values over time is random. This test combines information from consecutive samples. Only very large deviations from randomness can be detected when analyses are based on separate time intervals, and, if the trend in phylogenetic grouping or dispersion persists for more than one unit of time, the serial runs test will have more power to detect deviation from randomness.

## Results

### Phylogenetic structure

**Figure 1:**
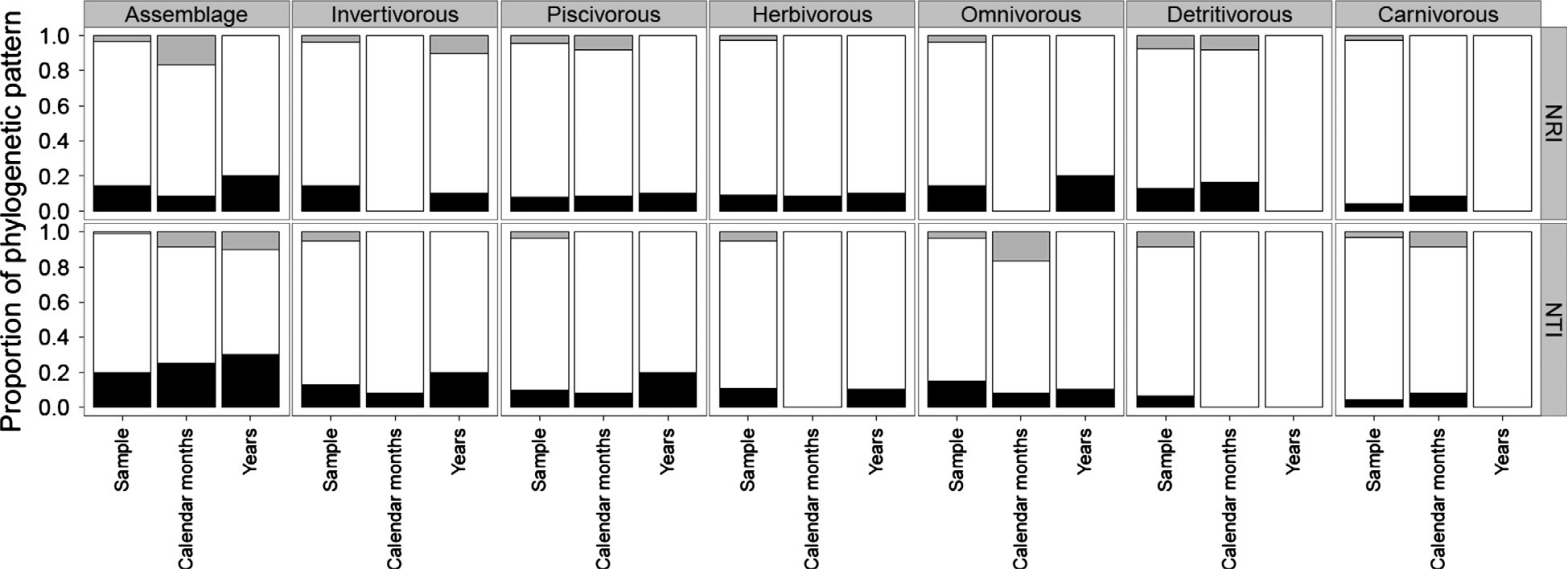
Proportion of phylogenetic patterns observed for assembly and functional groups. The bar-color indicates the phylogenetic patterns (Gray – Phylogenetic overdispersion; White – Random; and Black – Phylogenetic clustering).

The phylogenetic pattern in the whole assemblage and the functional groups varied widely among the three temporal scales (Appendix S4). For the whole assemblage and for each of the functional groups, the relative frequency of results that would be considered statistically significant in individual comparisons, indicating phylogenetic clustering or dispersion, depended on the temporal scale (Fig. 1). Overall, for all taxonomic groups, the patterns were more consistent with phylogenetic clustering than over dispersion.

### Trends in phylogenetic patterns over time

**Figure 2:**
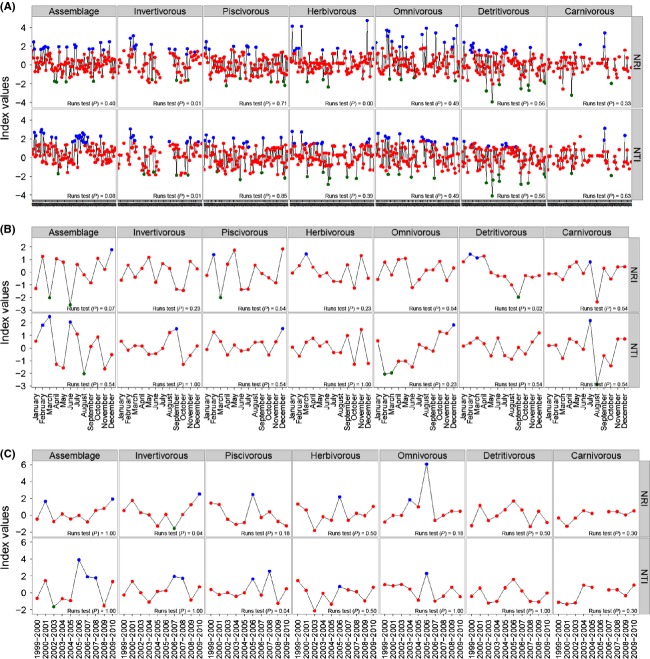
Distribution of index values for Net Relatedness Index (NRI) and Nearest Taxon Index (NTI) over time. The color indicates phylogenetic pattern (Blue – Phylogenetic clustering; Red – Random; and Green – Phylogenetic overdispersion). Discontinuities between points indicate the absence of functional group or occurrence of only one species from sample. (A) Sample; (B) Calendar months, and (C) Years.

The null hypothesis that the distribution of the values of NRI and NTI was random with respect to time was accepted in most tests (Fig. 2), but this conclusion depended on the temporal scale and index used. For the whole assemblage, the null hypothesis that the distribution of these values over time was random had little support. For other scales using the same index, the series were not distinguishable from random. At the scale of years (months pooled within each year), both indices always accepted the null hypothesis. For many functional groups, acceptance or rejection of the hypothesis of serial randomness also depended on the combination of scales and indices (Fig. 2).

We used the combination of index, null model, and species pool that we considered *a priori* most appropriate considering the natural history of the species. However, the use of other commonly used null models or combinations leads to qualitatively similar conclusions (Appendix S5–S10).

## Discussion

The temporal scale determined the frequency with which phylogenetic patterns were detected. Consequently, conclusions about the frequency with which biotic processes and environmental filters affect the local assembly do not depend only on taxonomic grouping and spatial scales (Cavender-Bares et al. 2006; Slingbsy and Verboom 2006; Swenson et al. 2006, 2007; Newton et al. 2007; Silver et al. 2012), but also depend on temporal scale used.

The ability to detect phylogenetic patterns depends on the relative importance of different processes that allow species to coexist (Kraft et al. 2007; Kembel 2009). Processes related to competitive exclusion are more readily detected using the NTI indices, while environmental filters are more easily detected by NRI (Kraft et al. 2007). However, there was no general pattern in our results for predictable differences between the indices. According to Kraft et al. (2007), the pool size of species that can contribute to the local assembly can affect the power of analyses, increasing rates of Type II error (failing to detect a pattern that is not random), or reducing the power to detect processes of competitive exclusion and increased power to detect environmental filters. Therefore, the phylogenetic pattern observed on one local scale may simply be related to the statistical power of the indices to detect nonrandom patterns and not due to processes of competitive exclusion or environmental filters. However, it is not possible to evaluate potential Type II errors only using field data. Our general conclusions did not depend on the choice of null models because other commonly used null models confirmed the dependence of conclusions on the index and species pool used (Appendix S11 and S12).

The differences that we attribute to the effects of temporal scales in phylogenetic patterns detected at a local scale may be related to the peculiarities of the environment and organisms we investigated. Abiotic and biotic factors related to spatial variability, seasonality (amplitude, duration, frequency, and regularity of the flood pulse), connectivity, ability to disperse (lateral and likely random movement of species), and colonization rates (Junk et al. 1989; Cox-Fernandes 1997; Winemiller and Jepsen 1998; Syms and Jones 2000; Petry et al. 2003; Arrington et al. 2005; Thomaz et al. 2007) vary over time, are factors known to influence fishes and others assemblages (e.g., Cottenie 2005; Alexander et al. 2012; Bie et al. 2012; Gothe et al. 2013), and can change the pool size of species, which affect the phylogenetic patterns detected in local assemblages.

While these analyses allow the assertion that all proposed patterns, and probably the processes inferred to cause them, apply to the fish assemblages in the floodplain, the assessment of the relative importance of these processes over time, and how they vary depending on the temporal scale and functional group studied, cannot be determined with the effort commonly used in studies of phylogenetic structure of assemblages. A better understanding of these mechanisms in local assemblages is fundamental to understanding the dynamics observed over time.

In this study, 10 years of data were necessary to show that phylogenetic structure varies widely over time on a local spatial scale, assemblage structure is hard to predict, and conclusions depend heavily on the time scale investigated. Studies of phylogenetic relationships have been used to explain various aspects of community and ecosystem functioning (Maherali and Klironomos 2007; Srivastava et al. 2012). However, we know little about the influence of rare species on the observed phylogenetic patterns (Gaston 2012; Mi et al. 2012), phylogenetic patterns of colonization and extinction vary widely (Cadotte and Strauss 2011), and the phylogenetic pattern observed in disturbed habitats may not be stable over time (Dinnage 2009; Helmus et al. 2010; Brundjerg et al. 2012). While it is possible that the strong dependence of the results on the choices of time scales and functional groups only applies to the floodplain fish assemblage we studied, we recommend that researchers evaluate the sensitivity of their results to temporal changes in assemblages, spatial variability, seasonality, connectivity, ability to disperse, and colonization rate before drawing general conclusions about the influence of environmental filtering and competitive exclusion in the assemblages they study. Perhaps, some of the instability in the results can be reduced by better field methods and larger sampling units that more closely reflect the interactions among species. However, in most cases, sampling is restrained by physical or logistic restraints, and previous studies are not available to evaluate the relative effectiveness of different sampling strategies.

## Supporting Information

**Appendix S1.** Study area. **Appendix S2.** Literature records that determined the guild classification of species based on their diet similarity. **Appendix S3**. List of species and guild classifications. **Appendix S4**. Distribution of index values for Net Relatedness Index (NRI) and Nearest Taxon Index (NTI) over three temporal scale. **Appendix S5**. Distribution of index values for Net Relatedness Index (NRI) and Nearest Taxon Index (NTI) over time with null model frequency. **Appendix S6**. Distribution of index values for Net Relatedness Index (NRI) and Nearest Taxon Index (NTI) over time with null model phylogeny pool. **Appendix S7**. Distribution of index values for Net Relatedness Index (NRI) and Nearest Taxon Index (NTI) over time with null model richness. **Appendix S8**. Distribution of index values for Net Relatedness Index (NRI) and Nearest Taxon Index (NTI) over time with null model sample pool. **Appendix S9**. Distribution of index values for Net Relatedness Index (NRI) and Nearest Taxon Index (NTI) over time with null model trial swap. **Appendix S10**. Distribution of index values for Net Relatedness Index (NRI) and Nearest Taxon Index (NTI) over time with null model taxa label. **Appendix S11**. Proportion of phylogenetic patterns observed for assembly and functional groups with NTI (Nearest Taxon Index). **Appendix S12**. Proportion of phylogenetic patterns observed for assembly and functional groups with NRI (Net Relatedness Index).
