# Supplementary material for: Phylogenetic community structure: temporal variation in fish assemblage
Source: Ecol Evol. 2014 May 1;4(11):2146–53. doi: 10.1002/ece3.1026 (PMC4201429; doi:10.1002/ece3.1026)
Supplement: Supplementary file 1 [file ece30004-2146-sd1.docx]

**Appendix 01 –** Study area

Fish were captured monthly in Catalão Lake , a floodplain lake, located at coordinates 3°10'04'' S and 59°54'45'' W, near the confluence of the Rio Negro and the Amazon River, during 10 years. Fish were captured with a standardized set of 10 gill nets, each10 meters long, with mesh size ranging from 24 to 120mm between opposite nodes, with a total area of ​​257m^2^, which was deployed monthly in the lake area during 24 hours. Nets were checked at 6 hour intervals (for more details of study area and sampling methods see Santos, Ferreira & Amadio 2008; Santos, Amadio & Ferreira 2010).


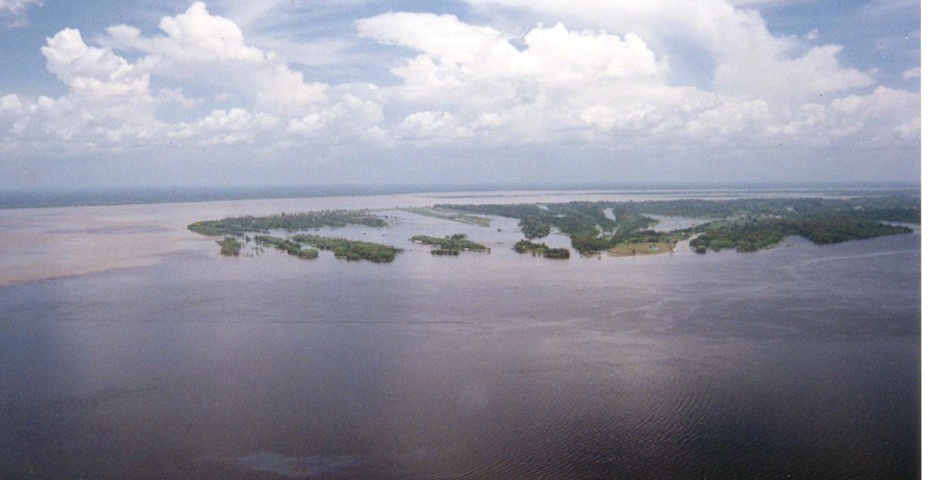


**Figure 01 -** The confluence of the Solimoes and Negro rivers and Catalão lake (Photo: Efrem Ferreira). The arrow in the photo shows the location of the lake, which is directly connected to the Amazon and Negro Rivers at high water.

**References**

Santos, R. F., Amadio, S., Ferreira, E., (2010) Patterns of energy allocation to reproduction in three Amazonian fish species. Neotropical Ichthyology, 8, 155-162.

Santos, R. F., Ferreira, E., Amadio, S. (2008) Effect of seasonality and trophic group on energy acquisition in Amazonian fish. Ecology of Freshwater Fish, 17, 340–348.

**Appendix 02** **–** Literature records that determined the guild classification of species based on their diet similarity.

Albrecht, M. (2005) Estrutura trófica da ictiofauna do rio Tocantins na região sob influência da usina hidrelétrica Serra da Mesa, Brasil Central. PhD thesis, Universidade Federal do Rio de Janeiro, Rio de Janeiro.

Almeida, R.G. (1984) Biologia alimentar de três espécies de Triportheus (Pisces: Characoidei, Characidae) do lago Castanho, Amazonas. Acta Amazonica, 14, 48-76.

Aragão, L.P. (1981) Desenvolvimento embrionário e larval, alimentação e reprodução do aruanã, Osteoglossum bicirrhosum Vandelli 1829, do Lago Janauacá – Amazonas, Brasil. MSc thesis, Instituto Nacional de Pesquisas da Amazônia, Manaus.

Carvalho, F. M. (1979) Estudo da alimentação, desenvolvimento dos ovários e composição química de Hypophthalmus edentatus (Spix, 1829) e Potamorhina pristigaster (Steindachner, 1978) do lago do Castanho, AM, Brasil. MSc thesis, Instituto Nacional de Pesquisas da Amazônia, Manaus.

Correa, S. (2005) Comparison of fish assemblages in flooded forest versus floating meadows habitats of an upper amazon floodplain (Pacaya Samiria National Reserve, Peru). PhD thesis, University of Florida, Florida.

Corredor, M. C. F. (2004) Influência das variações temporais da disponibilidade relativa de habitats sobre a comunidade de peixes em um lago de várzea da Amazônia Central. MSc thesis, Instituto Nacional de Pesquisas da Amazônia, Manaus.

Ferreira, E., Zuanon, J. & Santos, G. 1998. Peixes comerciais do médio rio Amazonas: região de Santarém, Pará. IBAMA, Brasília.

Godoi, D. (2008) Diversidade e hábitos alimentares de peixes de afluentes do rio Teles Pires, drenagem do rio Tapajós, bacia Amazônica. PhD thesis. Universidade Estadual Paulista, Jaboticabal.

Goulding, M. (1980) The Fishes and the Forest: Explorations in Amazonian Natural History. University of California Press, Los Angeles.

Goulding, M., Carvalho, M. & Ferreira, E. (1988) Rio Negro, rich life in poor water: Amazonian diversity and food chain ecology as seen through fish communities. SPB Academic Publishing, Amsterdam.

Honda, E. (1972) Contribuição ao conhecimento da biologia de peixes do Amazonas. I – Alimentação de Geophagus. Acta Amazonica, 2,81-88.

Lima, J. D. (2003) Diversidade, estrutura trófica da ictiofauna e condições limnológicas em um lago na planície inundável do Rio das Mortes – MT. MSc thesis, Universidade Federal do Mato Grosso, Cuiabá.

Merigoux, S. & Ponton, D. (1998) Body shape, diet and ontogenetic diet shifts in young ﬁsh of the Sinnamary River, French Guiana, South America. Journal of Fish Biology, 52,556–569.

Mérona, B., Santos, G. M. & Almeida, R. G. (2001) Short term effects of Tucuruí Dam (Amazonia, Brazil) on the trophic organization of fish communities. Environmental Biology of Fishes, 60,375-392.

Moreira, S. S. (2004) Relações entre o ciclo hidrológico, atividade alimentar e táticas reprodutivas de quatro espécies de peixes na área do Catalão, Amazônia Central. MSc thesis, Instituto Nacional de Pesquisas da Amazônia, Manaus.

Paixão, I. M. P. (1980) Estudo da alimentação e reprodução de Mylossoma duriventris Cuvier, 1818 (Pisces, Characoides), do Lago Janauacá, AM, Brasil. MSc thesis, Instituto Nacional de Pesquisas da Amazônia, Manaus.

Pouilly, M., Yunoki, T., Rosales, C. & Torres, L.(2004) Trophic structure of fish assemblages from Mamoré river floodplain lakes (Bolivia). Ecology of Freshwater Fish 13,245-257.

Py-Daniel, L. H. R. (1984) Sistemática dos Loricariidae (Ostariophysi,Siluroidei) do complexo de lagos do Janauacá, Amazonas e aspectos da sua biologia e ecologia. MSc thesis, Instituto Nacional de Pesquisa da Amazônia, Manaus.

Ropke, C. (2008) Estrutura trófica das assembleias de peixes em biótopos de herbáceas nos rios Araguaia (Tocantis) e Trombetas (Pará), Brasil. MSc thesis, Instituto Nacional de Pesquisas da Amazônia, Manaus.

Santos, G. M. (1979) Estudos da alimentação, reprodução e aspectos da sistemática de Schizodon fasciatus Agassiz, 1829, Rhytiodus microlepis Kner, 1859 e Rhytiodus argenteofuscus Kner, 1859 do lago Janauacá-AM, Brasil. MSc thesis, Instituto Nacional de Pesquisas da Amazônia, Manaus.

Santos, G. M. (1981) Estudos de alimentação e hábitos alimentares de Schizodon fasciatus Agassiz, 1829, Rhytiodus microlepis Kner, 1859 e Rhytiodus argenteofuscus Kner, 1859, do Lago Janauacá-Am. (Osteichthyes, Characoidei, Anostomidae). Acta Amazonica, 11,267-284.

Santos, G. M. (1982) Caracterização, hábitos alimentares e reprodutivos de quatro espécies de “aracus” e considerações ecológicas sobre o grupo no lago Janaucá-AM. (Osteichthyes, Characoidei, Anostomidae). Acta Amazonica, 12,713-739.

Santos, G. M. (1991) Pesca e ecologia dos peixes de Rondônia. PhD thesis, Instituto Nacional de Pesquisas da Amazônia, Manaus.

Santos, G. M., Ferreira, E. & Zuanon, J. (2006) Peixes comerciais de Manaus. Pro Várzea, Manaus.

Silva, C., Ferreira, E. & Deus, C. (2008) Dieta de cinco espécies de Hemiodontidae (Teleostei, Characiformes) na área de influência do reservatório de Balbina, rio Uatumã, Amazonas, Brasil. Iheringia. Série Zoologia, 98,464-468.

Silva, C. (2006) Dieta da comunidade de peixes na área de influência da UHE de Balbina - rio Uatumã, Amazonas, Brasil. MSc thesis, Instituto Nacional de Pesquisas da Amazônia, Manaus.

Soares, M., Costa, E., Souza, F., Anjos, H., Yamamoto, K. & Freitas, C. (2008) Peixes de lagos do Médio Rio Solmões, 2 ed rev. Instituto I – Piatam, Manaus.

Yamamoto, K. C., Soares, M. & Freitas, C. (2004) Alimentação de Triportheus angulatus (Spix & Agassiz, 1829) no lago Camaleão, Manaus, AM, Brasil. Acta Amazonica, 34, 653-659.

Zuanon, J. (1990) Aspectos da biologia, ecologia e pesca de grandes bagres (Pisces: Siluriformes, Siluroidei) na área da ilha de Marchantaria – rio Solimões, AM. MSc thesis. Instituto Nacional de Pesquisas da Amazônia, Manaus.

**Appendix 03** - List of species and guild classifications

| **Species** | **Guild** | | **Family** | | **Order** | |
| --- | --- | --- | --- | --- | --- | --- |
| Acarichthys heckelii (Müller & Troschel, 1849) | Invertivorous | | Cichlidae | | Perciformes | |
| Acaronia nassa (Heckel, 1840) | Piscivorous | | Cichlidae | | Perciformes | |
| Acestrorhynchus falcatus (Bloch, 1794) | Piscivorous | | Acestrorhynchidae | | Characiformes | |
| Acestrorhynchus falcirostris (Cuvier, 1819) | Piscivorous | | Acestrorhynchidae | | Characiformes | |
| Acestrorhynchus microlepis (Jardine, 1841) | Piscivorous | | Acestrorhynchidae | | Characiformes | |
| Aequidens tetramerus (Heckel, 1840) | Omnivorous | | Cichlidae | | Perciformes | |
| Agamyxis pectinifrons (Cope, 1870) | Invertivorous | | Doradidae | | Siluriformes | |
| Ageneiosus atronasus Eigenmann & Eigenmann, 1888 | Unidentified | | Auchenipteridae | | Siluriformes | |
| Ageneiosus brevis Steindachner, 1881 | Carnivorous | | Auchenipteridae | | Siluriformes | |
| Ageneiosus inermis (Linnaeus, 1766) | Carnivorous | | Auchenipteridae | | Siluriformes | |
| Ageneiosus piperatus (Eigenmann, 1912) | Invertivorous | | Auchenipteridae | | Siluriformes | |
| Ageneiosus vittatus Steindachner, 1908 | Invertivorous | | Auchenipteridae | | Siluriformes | |
| Ageneiosus ucayalensis Castelnau, 1855 | Invertivorous | | Auchenipteridae | | Siluriformes | |
| Anadoras grypus (Cope, 1872) | Omnivorous | | Doradidae | | Siluriformes | |
| Ancistrus dolichopterus Kner, 1854 | Detritivorous | | Loricariidae | | Siluriformes | |
| Ancistrus sp.1 Kner, 1854 | Unidentified | | Loricariidae | | Siluriformes | |
| Ancistrus sp.2 Kner, 1854 | Unidentified | | Loricariidae | | Siluriformes | |
| Anostomoides laticeps (Eigenmann, 1912) | Omnivorous | | Anostomidae | | Characiformes | |
| Astronotus crassipinnis (Heckel, 1840) | Omnivorous | | Cichlidae | | Perciformes | |
| Astronotus ocellatus (Agassiz, 1831) | Omnivorous | | Cichlidae | | Perciformes | |
| Auchenipterichthys longimanus (Günther, 1864) | Herbivorous | | Auchenipteridae | | Siluriformes | |
| Auchenipterichthys thoracatus (Kner, 1858) | Omnivorous | | Auchenipteridae | | Siluriformes | |
| Auchenipterus britskii Ferraris & Vari, 1999 | Invertivorous | | Auchenipteridae | | Siluriformes | |
| Auchenipterus nuchalis (Spix & Agassiz, 1829) | Invertivorous | | Auchenipteridae | | Siluriformes | |
| Biotodoma cupido (Heckel, 1840) | Omnivorous | | Cichlidae | | Perciformes | |
| Boulengerella cuvieri (Spix & Agassiz, 1829) | Piscivorous | | Ctenoluciidae | | Characiformes | |
| Boulengerella lucius (Cuvier, 1816) | Piscivorous | | Ctenoluciidae | | Characiformes | |
| Boulengerella maculata (Valenciennes, 1850) | Piscivorous | | Ctenoluciidae | | Characiformes | |
| Catoprion mento (Cuvier, 1819) | Piscivorous | | Characidae | | Characiformes | |
| Centromochlus heckelii (De Filippi, 1853) | Invertivorous | | Auchenipteridae | | Siluriformes | |
| Chaetobranchopsis orbicularis (Steindachner, 1875) | Unidentified | | Cichlidae | | Perciformes | |
| Chaetobranchus flavescens Heckel, 1840 | Invertivorous | | Cichlidae | | Perciformes | |
| Charax sp. Gronow 1763 | Unidentified | | Characidae | | Characiformes | |
| Cichla monoculus Spix & Agassiz, 1831 | Piscivorous | | Cichlidae | | Perciformes | |
| Cichlasoma amazonarum Kullander, 1983 | Detritivorous | | Cichlidae | | Perciformes | |
| Colossoma macropomum (Cuvier, 1816) | Herbivorous | | Characidae | | Characiformes | |
| Crenicichla cincta Regan, 1905 | Carnivorous | | Cichlidae | | Perciformes | |
| Crenicichla inpa Ploeg, 1991 | Unidentified | | Cichlidae | | Perciformes | |
| Crenicichla lugubris Heckel, 1840 | Piscivorous | | Cichlidae | | Perciformes | |
| Crenicichla reticulata (Heckel, 1840) | Unidentified | | Cichlidae | | Perciformes | |
| Curimata inornata Vari, 1989 | Detritivorous | | Curimatidae | | Characiformes | |
| Curimata knerii Steindachner, 1876 | Detritivorous | | Curimatidae | | Characiformes | |
| Curimata vittata (Kner, 1858) | Detritivorous | | Curimatidae | | Characiformes | |
| Curimatella alburna (Müller & Troschel, 1844) | Detritivorous | | Curimatidae | | Characiformes | |
| Curimatella meyeri (Steindachner, 1882) | Detritivorous | | Curimatidae | | Characiformes | |
| Cynodon gibbus (Agassiz, 1829) | Piscivorous | | Cynodontidae | | Characiformes | |
| Cyphocharax plumbeus (Eigenmann & Eigenmann, 1889) | Detritivorous | | Curimatidae | | Characiformes | |
| Cyphocharax spiluropsis (Eigenmann & Eigenmann, 1889) | Detritivorous | | Curimatidae | | Characiformes | |
| Dianema longibarbis Cope, 1872 | Omnivorous | | Callichthyidae | | Siluriformes | |
| Dianema urostriatum (Miranda Ribeiro, 1912) | Unidentified | | Callichthyidae | | Siluriformes | |
| Doras punctatus Kner, 1853 | Invertivorous | | Doradidae | | Siluriformes | |
| Eigenmannia limbata (Schreiner & Miranda Ribeiro, 1903) | Invertivorous | | Sternopygidae | | Gymnotiformes | |
| Epapterus dispilurus Cope, 1878 | Invertivorous | | Auchenipteridae | | Siluriformes | |
| Geophagus proximus (Castelnau, 1855) | Omnivorous | | Cichlidae | | Perciformes | |
| Gymnotus carapo Linnaeus, 1758 | Omnivorous | | Gymnotidae | | Gymnotiformes | |
| Hassar sp. Eigenmann & Eigenmann, 1858 | Unidentified | | Doradidae | | Siluriformes | |
| Hemidoras morrisi Eigenmann, 1925 | Unidentified | | Doradidae | | Siluriformes | |
| Hemidoras stenopeltis (Kner, 1855) | Detritivorous | | Doradidae | | Siluriformes | |
| Hemisorubim platyrhynchos (Valenciennes, 1840) | Piscivorous | | Pimelodidae | | Siluriformes | |
| Heros efasciatus Heckel, 1840 | Omnivorous | | Cichlidae | | Perciformes | |
| Hoplias malabaricus (Bloch, 1794) | Piscivorous | | Erythrinidae | | Characiformes | |
| Hoplosternum littorale (Hancock, 1828) | Invertivorous | | Callichthyidae | | Siluriformes | |
| Hydrolycus scomberoides (Cuvier, 1819) | Piscivorous | | Cynodontidae | | Characiformes | |
| Hypostomus emarginatus Valenciennes, 1840 | Detritivorous | | Loricariidae | | Siluriformes | |
| Hypostomus plecostomus (Linnaeus, 1758) | Unidentified | | Loricariidae | | Siluriformes | |
| Hypselecara temporalis (Günther, 1862) | Omnivorous | | Cichlidae | | Perciformes | |
| Ilisha amazonica (Miranda Ribeiro, 1920) | Invertivorous | | Pristigasteridae | | Clupeiformes | |
| Laemolyta proxima (Garman, 1890) | Omnivorous | | Anostomidae | | Characiformes | |
| Laemolyta taeniata (Kner, 1858) | Omnivorous | | Anostomidae | | Characiformes | |
| Leiarius marmoratus (Gill, 1870) | Carnivorous | | Pimelodidae | | Siluriformes | |
| Leporinus amazonicus Santos & Zuanon, 2008 | Unidentified | | Anostomidae | | Characiformes | |
| Leporinus fasciatus (Bloch, 1794) | Omnivorous | | Anostomidae | | Characiformes | |
| Leporinus friderici (Bloch, 1794) | Omnivorous | | Anostomidae | | Characiformes | |
| Leporinus trifasciatus Steindachner, 1876 | Omnivorous | | Anostomidae | | Characiformes | |
| Loricariichthys acutus (Valenciennes, 1840) | Omnivorous | | Loricariidae | | Siluriformes | |
| Loricariichthys maculatus (Bloch, 1794) | Omnivorous | | Loricariidae | | Siluriformes | |
| Loricariichthys nudirostris (Kner, 1853) | Omnivorous | | Loricariidae | | Siluriformes | |
| Loricariichthys platymetopon Isbrücker & Nijssen, 1979 | Omnivorous | | Loricariidae | | Siluriformes | |
| Lycengraulis batesii (Günther, 1868) | Unidentified | | Engraulidae | | Clupeiformes | |
| Lycengraulis grossidens (Spix & Agassiz, 1829) | Piscivorous | | Engraulidae | | Clupeiformes | |
| Megalechis picta (Müller & Troschel, 1849) | Unidentified | | Callichthyidae | | Siluriformes | |
| Megalodoras uranoscopus (Eigenmann & Eigenmann, 1888) | Omnivorous | | Doradidae | | Siluriformes | |
| Mesonauta festivus (Heckel, 1840) | Omnivorous | | Cichlidae | | Perciformes | |
| Mesonauta insignis (Heckel, 1840) | Detritivorous | | Cichlidae | | Perciformes | |
| Metynnis argenteus Ahl, 1923 | Herbivorous | | Characidae | | Characiformes | |
| Metynnis hypsauchen (Müller & Troschel, 1844) | Omnivorous | | Characidae | | Characiformes | |
| Metynnis lippincotianus (Cope, 1870) | Omnivorous | | Characidae | | Characiformes | |
| Metynnis luna Cope, 1878 | Invertivorous | | Characidae | | Characiformes | |
| Myloplus rubripinnis (Müller & Troschel, 1844) | Herbivorous | | Characidae | | Characiformes | |
| Myloplus torquatus (Kner 1858) | Herbivorous | | Characidae | | Characiformes | |
| Mylossoma aureum (Spix & Agassiz, 1829) | Herbivorous | | Characidae | | Characiformes | |
| Mylossoma duriventre (Cuvier, 1818) | Herbivorous | | Characidae | | Characiformes | |
| Nemadoras elongatus (Boulenger, 1898) | Omnivorous | | Doradidae | | Siluriformes | |
| Nemadoras hemipeltis (Eigenmann, 1925) | Invertivorous | | Doradidae | | Siluriformes | |
| Nemadoras humeralis (Kner, 1855) | Invertivorous | | Doradidae | | Siluriformes | |
| Oxydoras eigenmanni Boulenger, 1895 | Unidentified | | Doradidae | | Siluriformes | |
| Oxydoras niger (Valenciennes, 1821) | Detritivorous | | Doradidae | | Siluriformes | |
| Parapteronotus hasemani (Ellis, 1913) | Invertivorous | | Apteronotidae | | Gymnotiformes | |
| Parauchenipterus galeatus (Linnaeus, 1766) | Omnivorous | | Auchenipteridae | | Siluriformes | |
| Parauchenipterus porosus (Eigenmann & Eigenmann 1888) | Omnivorous | | Auchenipteridae | | Siluriformes | |
| Pellona castelnaeana Valenciennes, 1847 | Piscivorous | | Pristigasteridae | | Clupeiformes | |
| Pellona flavipinnis (Valenciennes, 1837) | Carnivorous | | Pristigasteridae | | Clupeiformes | |
| Phractocephalus hemioliopterus (Bloch & Schneider, 1801) | Piscivorous | | Pimelodidae | | Siluriformes | |
| Piaractus brachypomus (Cuvier, 1818) | Herbivorous | | Characidae | | Characiformes | |
| Pimelodella sp. Eigenmann & Eigenmann, 1888 | Unidentified | | Heptapteridae | | Siluriformes | |
| Potamorhina altamazonica (Cope, 1878) | Detritivorous | | Curimatidae | | Characiformes | |
| Potamorhina latior (Spix & Agassiz, 1829) | Detritivorous | | Curimatidae | | Characiformes | |
| Potamorhina pristigaster (Steindachner, 1876) | Detritivorous | | Curimatidae | | Characiformes | |
| Prochilodus nigricans Spix & Agassiz, 1829 | Herbivorous | | Prochilodontidae | | Characiformes | |
| Psectrogaster amazonica Eigenmann & Eigenmann, 1889 | Detritivorous | | Curimatidae | | Characiformes | |
| Psectrogaster rutiloides (Kner, 1858) | Detritivorous | | Curimatidae | | Characiformes | |
| Pseudoplatystoma punctifer (Castelnau,1855) | Unidentified | | Pimelodidae | | Siluriformes | |
| Pseudoplatystoma tigrinum (Valenciennes, 1840) | Carnivorous | | Pimelodidae | | Siluriformes | |
| Pterodoras granulosus (Valenciennes, 1821) | Herbivorous | | Doradidae | | Siluriformes | |
| Pterodoras lentiginosus (Eigenmann 1917) | Omnivorous | | Doradidae | | Siluriformes | |
| Pterophyllum scalare (Schultze, 1823) | Invertivorous | | Cichlidae | | Perciformes | |
| Pterygoplichthys pardalis (Castelnau, 1855) | Detritivorous | | Loricariidae | | Siluriformes | |
| Pygocentrus nattereri Kner, 1858 | Piscivorous | | Characidae | | Characiformes | |
| Rhamphichthys marmoratus Castelnau, 1855 | Omnivorous | | Rhamphichthyidae | | Gymnotiformes | |
| Rhamphichthys rostratus (Linnaeus, 1766) | Invertivorous | | Rhamphichthyidae | | Gymnotiformes | |
| Rhamphichthys vulpinus (?) | Unidentified | | Rhamphichthyidae | | Gymnotiformes | |
| Rhaphiodon vulpinus Spix & Agassiz, 1829 | Piscivorous | | Cynodontidae | | Characiformes | |
| Rhytiodus argenteofuscus Kner, 1858 | Herbivorous | | Anostomidae | | Characiformes | |
| Rhytiodus microlepis Kner, 1858 | Herbivorous | | Anostomidae | | Characiformes | |
| Roeboides affinis (Günther, 1864) | Piscivorous | | Characidae | | Characiformes | |
| Roeboides myersii Gill, 1870 | Carnivorous | | Characidae | | Characiformes | |
| Satanoperca auticeps (Heckel, 1840) | Omnivorous | | Cichlidae | | Perciformes | |
| Satanoperca jurupari (Heckel, 1840) | Omnivorous | | Cichlidae | | Perciformes | |
| Schizodon fasciatus Spix & Agassiz, 1829 | Herbivorous | | Anostomidae | | Characiformes | |
| Semaprochilodus insignis (Jardine, 1841) | Detritivorous | | Prochilodontidae | | Characiformes | |
| Semaprochilodus taeniurus (Valenciennes, 1821) | Detritivorous | | Prochilodontidae | | Characiformes | |
| Serrasalmus altispinis Merckx, Jégu & Santos, 2000 | Piscivorous | | Characidae | | Characiformes | |
| Serrasalmus compressus Jégu, Leão & Santos, 1991 | Unidentified | | Characidae | | Characiformes | |
| Serrasalmus eigenmanni Norman, 1929 | Piscivorous | | Characidae | | Characiformes | |
| Serrasalmus elongatus Kner, 1858 | Piscivorous | | Characidae | | Characiformes | |
| Serrasalmus maculatus Kner, 1858 | Omnivorous | | Characidae | | Characiformes | |
| Serrasalmus rhombeus (Linnaeus, 1766) | Carnivorous | | Characidae | | Characiformes | |
| Serrasalmus robertsoni (?) | Carnivorous | | Characidae | | Characiformes | |
| Serrasalmus serrulatus (Valenciennes, 1850) | Herbivorous | | Characidae | | Characiformes | |
| Sorubim elongatus Littmann, Burr, Schmidt & Isern, 2001 | Carnivorous | | Pimelodidae | | Siluriformes | |
| Sorubim lima Sorubim lima (Bloch & Schneider, 1801) | Invertivorous | | Pimelodidae | | Siluriformes | |
| Sorubim maniradii Littmann, Burr & Buitrago-Suarez, 2001 | Unidentified | | Pimelodidae | | Siluriformes | |
| Steindachnerina bimaculata (Steindachner, 1876) | Detritivorous | | Curimatidae | | Characiformes | |
| Steindachnerina leucisca (Günther, 1868) | Detritivorous | | Curimatidae | | Characiformes | |
| Sternarchorhynchus roseni Mago-Leccia, 1994 | Unidentified | | Apteronotidae | | Gymnotiformes | |
| Sternopygus macrurus (Bloch & Schneider, 1801) | Invertivorous | | Sternopygidae | | Gymnotiformes | |
| Sturisoma sp. Swainson, 1838 | Unidentified | | Loricariidae | | Siluriformes | |
| Tatia intermedia (Steindachner, 1877) | Invertivorous | | Auchenipteridae | | Siluriformes | |
| Trachydoras nattereri (Steindachner, 1881) | Invertivorous | | Doradidae | | Siluriformes | |
| Uaru amphiacanthoides Heckel, 1840 | Herbivorous | | Cichlidae | | Perciformes | |
| Zungaro zungaro (Humboldt, 1821) | Carnivorous | Pimelodidae | | Siluriformes | |  |
|  |  |  | |  | |  |

**Appendix 04** – Distribution of index values for Net Relatedness Index (NRI) and Nearest Taxon Index (NTI) over three temporal scale. The color indicates type of phylogenetic pattern ( Blue – Phylogenetic clustering; Red – Random; and Green – Phylogenetic overdispersion).

1.
2.
3.

**Appendix 05 -** Distribution of index values for Net Relatedness Index (NRI) and Nearest Taxon Index (NTI) over time with null model Frequency. The color indicates phylogenetic pattern ( Blue – Phylogenetic clustering; Red – Random; and Green – Phylogenetic overdispersion). Discontinuities between points indicate the absence of functional group or occurrence of only one species from sample. A) Sample; B) Calendar months and C) Years.

1.
2.
3.

**Appendix 06 -** Distribution of index values for Net Relatedness Index (NRI) and Nearest Taxon Index (NTI) over time with null model Phylogeny pool. The color indicates phylogenetic pattern ( Blue – Phylogenetic clustering; Red – Random; and Green – Phylogenetic overdispersion). Discontinuities between points indicate the absence of functional group or occurrence of only one species from sample. A) Sample; B) Calendar months and C) Years.

1.
2.

**Appendix 07 -** Distribution of index values for Net Relatedness Index (NRI) and Nearest Taxon Index (NTI) over time with null model Richness. The color indicates phylogenetic pattern ( Blue – Phylogenetic clustering; Red – Random; and Green – Phylogenetic overdispersion). Discontinuities between points indicate the absence of functional group or occurrence of only one species from sample. A) Sample; B) Calendar months and C) Years.

1.
2.
3.

**Appendix 08 -** Distribution of index values for Net Relatedness Index (NRI) and Nearest Taxon Index (NTI) over time with null model Sample pool. The color indicates phylogenetic pattern ( Blue – Phylogenetic clustering; Red – Random; and Green – Phylogenetic overdispersion). Discontinuities between points indicate the absence of functional group or occurrence of only one species from sample. A) Sample; B) Calendar months and C) Years.

1.
2.
3.

**Appendix 09 -** Distribution of index values for Net Relatedness Index (NRI) and Nearest Taxon Index (NTI) over time with null model Trial swap. The color indicates phylogenetic pattern ( Blue – Phylogenetic clustering; Red – Random; and Green – Phylogenetic overdispersion). Discontinuities between points indicate the absence of functional group or occurrence of only one species from sample. A) Sample; B) Calendar months and C) Years.

1.
2.
3.

**Appendix 10 -** Distribution of index values for Net Relatedness Index (NRI) and Nearest Taxon Index (NTI) over time with null model Taxa label. The color indicates phylogenetic pattern ( Blue – Phylogenetic clustering; Red – Random; and Green – Phylogenetic overdispersion). Discontinuities between points indicate the absence of functional group or occurrence of only one species from sample. A) Sample; B) Calendar months and C) Years.

**Appendix 11 –** Proportion of phylogenetic patterns observed for assembly and functional groups with NTI (Nearest Taxon Index) . The bar-color indicates the phylogenetic patterns (Gray - Phylogenetic overdispesion; White - Random; and Black - Phylogenetic clustering). B) NRI (Net Relatedness Index). Model-1 (Independent swap), Model-2 (Frequency), Model-3 (Trial swap), Model-4 (Sample pool), Model-5 (Taxa label), Model-6 (Phylogeny pool), Model-7 (Richness).

**Appendix 12 -** Proportion of phylogenetic patterns observed for assembly and functional groups with NRI (Net Relatedness Index). The bar-color indicates the phylogenetic patterns (Gray - Phylogenetic overdispesion; White - Random; and Black - Phylogenetic clustering). Model-1 (Independent swap), Model-2 (Frequency), Model-3 (Trial swap), Model-4 (Sample pool), Model-5 (Taxa label), Model-6 (Phylogeny pool), Model-7 (Richness).
